# Comment on ‘Cytoreductive surgery plus hyperthermic intraperitoneal chemotherapy versus R0 resection for resectable colorectal cancer with peritoneal metastases and low peritoneal cancer index scores: A collaborative observational study from Korea and Japan’

**DOI:** 10.1097/JS9.0000000000000932

**Published:** 2023-11-27

**Authors:** Bo Pei, Jia Zhu, Lin Lai

**Affiliations:** a Department of Oncology, Central Hospital of Enshi Tujia and Miao Autonomous Prefecture, Enshi Clinical College of Wuhan University; b Department of Clinical Medicine, Hubei Enshi College, Enshi, People’s Republic of China

Colorectal cancer (CRC) is the third most common cancer and the third most common cause of cancer death in both men and women in the United States^1^. Colorectal cancer with peritoneal metastasis (CPM) is present in 5–15% of cases of CRC, and it has been considered as the terminal stage with disappointing outcomes^2^. Without effective and timely treatment, the median survival time of CPM is only 6 months^3^.

CPM was considered incurable, and palliative systemic chemotherapy was the only appropriate option in a not-so-distant past. Currently, both the National Comprehensive Cancer Network (NCCN) guidelines and the European Society of Medical Oncology (ESMO) guidelines recommend cytoreductive surgery (CRS) plus intraperitoneal hyperperfusion chemotherapy (HIPEC) for the treatment of mild to moderate CPM. However, the value of HIPEC in the integrated treatment system is one of the key issues that has been debated for a long time^4^. In this paper, the authors analyzed the collaborative observational study in Korea and Japan to demonstrate that CRS plus HIPEC can be considered as local control therapy for colorectal cancer patients with limited peritoneal seeding. Although the results of this article have significant guiding implications for clinical practice, we still have some concerns about this conclusion.

First, this study only selected patients treated with CRS+HIPEC in South Korea to compare with patients treated with R0 resection in Japan. Are there no patients treated with R0 resection in South Korea and CRS+HIPEC in Japan? There may be selection bias. The use of HIPEC drugs for colon cancer is also different in different regions. In the United States, mitomycin-based Sugarbaker protocol is administered, while in European countries, oxaliplatin-based Dutch protocol is adopted, and the chemotherapy dosage range is extremely broad. The mitomycin regimen was administered in this study, but the number of chemotherapy cycles was not specified. The therapeutic value of HIPEC for CPM was questioned, which had an effect on the application of the conclusion. The academic community generally believed that the chemotherapy regimen of HIPEC should be further explored, and combining different existing drugs and new targeted drug development became the future direction of HIPEC therapy.

Second, patients who underwent surgery for both simultaneous and metachronous CPM were included in this study, and no subgroup analysis was performed. The incidence and prognosis of simultaneous and metachronous CPM are different, and there is evidence that compared with metachronous peritoneal metastases (PMs), simultaneous PMs often indicate a wider lesion range and worse prognosis^5^. Therefore, the lack of subgroup analysis of the relationship between simultaneous and metachronous PMs and the primary and secondary endpoints in this study will significantly affect the accuracy of the conclusion.

In addition, the basis for the selection of peritoneal cancer index (PCI) scores was not explained. PCI score is currently considered to be the best indicator for screening CRS patients and is also a reliable and effective clinical tool for evaluating tumor burden and PM implantation extent. PCI<20 is often considered to be the surgical indication of CRS for CPM, while CRS is not recommended for PCI>20^6^. In 2021, Quénet *et al*.^7^ compared the treatment difference between CRS plus HIPEC and CRS alone and found that the survival time of patients after the two treatments were 41.2 months and 41.7 months, respectively, with no statistical significance. Patients with a PCI score of 11–15 can benefit from HIPEC. In the present study, why PCI<6 was chosen as the cut-off value? The basis for PCI<6 as a cut-off value needs further clarification.

Collectively, we appreciate the authors’ efforts in applying CRS+HIPEC to the treatment of low PCI score resectable colorectal peritoneal cancer, which provides strong evidence for the clinical practice of colorectal cancer with localized PM. However, CPM is a subtype of metastatic colorectal cancer with unique tumor biological behavior, which cannot be understood and treated with traditional therapeutic concepts. Individualized therapy and precision treatment should be considered in the era of molecular medicine, such as the precise selection of drugs based on gene mutations, advantages of PD-1 (programmed cell death protein 1) immunotherapy, drug sensitivity of the organoid model to improve treatment precision, and the key molecular pathological characteristics of consensus molecular subtypes 4 (CMS4) in CPM therapy. Hence, larger, international, multicenter prospective studies confirm that CRS plus HIPEC can achieve curative treatment with local control for colorectal cancer patients with limited peritoneal seeding.

## Ethical approval

Not applicable.

## Consent

Not applicable.

## Sources of funding

This study was supported by the Natural Science Foundation of Enshi Tujia and the Miao Autonomous Prefecture Government (D20220060).

## Author contribution

B.P.: writing and study design; J.Z.: data collections; L.L.: critical revision of the manuscript.

## Conflicts of interest disclosure

There are no conflicts of interest.

## Research registration unique identifying number (UIN)

This is a commentary, no UIN is required.

## Guarantor

Bo Pei.

## Provenance and peer review

No, our paper was not invited.

## Data availability statement

Not applicable.
